# A NOVEL MULTIFREQUENCY GPS APP FOR STUDYING OLDER ADULTS: IMPROVING ACCURACY AND RELIABILITY OF DATA

**DOI:** 10.1093/geroni/igad104.2201

**Published:** 2023-12-21

**Authors:** Dinesh Mendhe, Stephanie Bergren, Yanping Jiang

**Affiliations:** Rutgers University New Brunswick, Piscataway, New Jersey, United States; Rutgers, the State University of New Jersey, New Brunswick, New Jersey, United States; Rutgers University, New Brunswick, New Jersey, United States

## Abstract

Acquiring global positioning system (GPS) data can help researchers understand the relationship between older adult’s location/activity and their health, but existing GPS data collection modes suffer from issues with precision, consistency, and acceptability. Some limitations of current GPS mobile apps are satellite service disruption, power saving mode interference, miscalibration of device’s compass, and user-error during data collection which result in missing or inaccurate data. We present a novel multilingual GPS mobile app and live administrative dashboard to address these limitations. The app performs triangulation between GPS satellite data, nearest cellular tower, and WiFi router location data which are processed through a unique GPS algorithm with a mean error of only 1.5 meters. Since GPS data results in coordinates commonly reverse geo-coded to place-based datasets, the accuracy is critical in determining place type or other characteristics. The app is additionally designed with redundancy and fault tolerance in mind to run continuously in the background of a user’s smartphone, so there is no data loss from accidentally closing the application or restarting the phone. Moreover, this new application has a flexible sampling rate ranging from 1 second to 20 minutes. Sampling frequency flexibility can adjust the battery life and reduce the need to charge as frequently. Last, application programming interface (API) integrations allow for live data stream, which can improve monitoring and troubleshooting in the field. For research with older adult populations, these features can not only improve the accuracy of collected data but also increase the acceptability of collecting GPS data.

